# A Conservative Mutant Version of the Mrr1 Transcription Factor Correlates with Reduced Sensitivity to Fludioxonil in *Botrytis cinerea*

**DOI:** 10.3390/pathogens13050374

**Published:** 2024-04-30

**Authors:** Charleen Copier, Claudio Osorio-Navarro, Jonathan E. Maldonado, Jaime Auger, Herman Silva, Marcela Esterio

**Affiliations:** 1 Laboratorio de Fitopatología Frutal y Molecular, Departamento de Sanidad Vegetal, Facultad de Ciencias Agronómicas, Universidad de Chile, La Pintana, Santiago 8820808, Chile; charleencopier@uchile.cl (C.C.); anduin@ug.uchile.cl (C.O.-N.); jauger@uchile.cl (J.A.); 2 Laboratorio de Genómica Funcional y Bioinformática, Departamento de Producción Agrícola, Facultad de Ciencias Agronómicas, Universidad de Chile, La Pintana, Santiago 8820808, Chile; jonathan.maldonado@usach.cl; 3 Plant Molecular Biology Centre, Departamento de Biología, Facultad de Ciencias, Universidad de Chile, Ñuñoa, Santiago 7800003, Chile

**Keywords:** grey mold, Mrr1, *atrB*, *Vitis vinifera*, table grapes, fludioxonil sensitivity

## Abstract

Fludioxonil is a highly effective phenylpyrrole fungicide for controlling *Botrytis cinerea*. Although the field efficacy of fludioxonil remains high, *Botrytis cinerea* isolates with reduced sensitivity have been reported globally. The molecular target of fludioxonil still remains unknown; however, a mechanism of reduced sensitivity to fludioxonil underlies the overexpression of the ATP binding cassette (ABC) transporter AtrB in a dependent pathway of the Mrr1 transcription factor. Fludioxonil is a key player in controlling *B. cinerea* infection in table grapes in Chile. However, some isolates with a reduced sensitivity to fludioxonil were detected. This study observed endogenous *atrB* overexpression in Chilean isolates with reduced sensitivity to fludioxonil (*n* = 22) compared to the sensitive isolates (*n* = 10). All isolates increased the expression of *atrB* in a growth medium supplemented with fludioxonil (0.05 μg/mL). However, sensitive isolates showed lower *atrB* expression than those with reduced fludioxonil sensitivity. Remarkably, a mutant version of the transcription factor Mrr1 carrying 21 amino acid modifications was identified in all isolates with reduced sensitivity to fludioxonil. These changes alter the protein’s transcription factor domain and the C-terminal portion of the protein but not the Zn (2)-C6 fungal-type DNA-binding domain. These results suggest a direct relationship between the conserved and divergent mutant version of *mrr1* and sensitivity to fludioxonil. This study provides a new target for developing molecular diagnostic strategies to monitor *B. cinerea*’s sensitivity to fludioxonil in the field.

## 1. Introduction

*Botrytis cinerea* Pers.Fr., the causal agent of grey mold in table grapes, is one of the most widely distributed necrotrophic pathogens worldwide with an extensive host range, affecting more than 1400 plant species [1]. Economic losses associated with *B. cinerea* include several fruits, vegetables, and ornamental plants. The damage caused by this type of infection seriously affects the commercialization of table grapes in Chile and several other countries, resulting in economic losses of up to 30% or more in production [2]. In table grapes, the pathogen can infect different plant organs at various stages of development but is most destructive in mature or senescent tissues. Floral organs are highly susceptible to *B. cinerea* infection and often serve as the pathogen’s entry point during fruit development. *B. cinerea* can remain quiescent until the environment is appropriate and the host physiology changes, after which it rapidly rots the plant tissues [3,4,5,6,7,8]. In Chile and worldwide, table grape grey mold control relies on the preventive application of synthetic fungicides in the most infection-susceptible periods: flowering, and from veraison to harvest [9,10]. Chilean table grapes receive over eight fungicide applications per season to control disease. However, in some cases, the management of *B. cinerea* has not been entirely satisfactory. Intensive application of fungicides with the same molecular target causes reduced sensitivity in fungal populations, leading to poor *B. cinerea* control [11,12,13,14,15]. 

Fludioxonil, a phenylpyrrole fungicide, is a non-systemic analog of the antibiotic pyrrolnitrin produced by various *Pseudomonas* species [16]. Fludioxonil is highly efficient in controlling plant pathogens, including *B. cinerea* [17]. In the beginning, its use was mainly in the control of soil pathogens due to their sensitivity to light [18]; however, in the 1980s, Ciba-Geigy AG (now Syngenta AG) developed two light-resistant synthetic analogs, one of them fludioxonil, to be used in seed treatment and foliar applications [19]. This molecule was introduced into the world market in 1993 to be used pre- and post-harvest. Phenylpyrroles are believed to target the High Osmolarity Glycerol (HOG) stress response signal transduction pathway, although their mode of action is not fully understood [17,20]. The HOG pathway is required to manage osmotic and other abiotic stress and has been extensively studied in the fludioxonil-insensitive yeast *Saccharomyces cerevisiae*. The group III hybrid histidine kinase (HHK) is considered the osmosensor in the HOG pathway and the putative molecular target of phenylpyrroles. Indeed, HHK Os-1 expression from *Neurospora crassa* can reverse sensitivity to fludioxonil in fludioxonil-insensitive yeast *S. cerevisiae* [21]. Consistently, mutations in the *os-1* gene of *N. crassa* trigger the reduced sensitivity to fludioxonil [22,23]. *B. cinerea* laboratory-generated and field strains mutant in the *os-1* homolog have reduced sensitivity to fludioxonil [24,25,26,27]. Alternatively, fludioxonil indirectly affects HHK-dependent signaling by acting on triosephosphate isomerase (TPI) in yeast [28]. However, whether TPI is the primary target in other fungal species remains to be shown. In most cases, the reduced sensitivity to fludioxonil due to mutations in the *os-1* homolog seems to induce a substantial fitness penalty associated with decreased sporulation, sclerotia production, and pathogenicity. This phenomenon could cause low fludioxonil field resistance [29,30]. However, not all fludioxonil-resistant *B. cinerea* field isolates exhibit changes in Os-1 or other components of the HOG pathway. In addition, moderate and low resistance to fludioxonil can be achieved through a drug-efflux-based multidrug resistance (MDR) mechanism. MDR underlies the overexpression of plasma membrane transporters of low substrate specificity, reducing sensitivity to unrelated fungicide molecules [31,32,33]. In *B. cinerea*, MDR is related to the ATP binding cassette (ABC) transporter gene *atrB* overexpression [34]. The transcriptional regulation of *atrB* is convergent among various fungi and depends on the transcription factor Mrr1 [35]. Mutations in the *mrr1* gene result in two main phenotypes: MDR1, conferring low resistance and characterized by different point mutations in the *mrr1* gene [34,36,37,38], and MDR1h, conferring moderate-to-high resistance to fludioxonil and characterized by a 3-bp deletion mutation in *mrr1*, leading to an amino acid deletion of L497 [38,39,40].

We recently reported the first Chilean *B. cinerea* field isolates with reduced sensitivity to fludioxonil from table grapes [41]. The low frequency of these isolates is consistent with worldwide reports and correlates with the high efficacy of fludioxonil in table grape phytosanitary programs [17]. However, compared to isolates from the United States and China, Chilean isolates with low fludioxonil sensitivity exhibit little to no fitness cost [37,41,42,43]. 

The goal of this study was to characterize the molecular basis of the reduced sensitivity to fludioxonil in the *B. cinerea* Chilean isolates, comparing biological fitness parameters among *B. cinerea* sensitive and *B. cinerea* with reduced sensitivity to fludioxonil, the expression levels of the *atrB* gene, and the genomic analysis of the *mrr1* and *atrB* genes.

## 2. Materials and Methods

### 2.1. Fungal Isolates, Culture Media, and Fungicide

Isolates of *B. cinerea* sensitive and with reduced sensitivity to fludioxonil were previously described [41]. All isolates were recovered from table grape cv. ‘Thompson Seedless’ naturally infected flowers, which were sampled during the 2016/2017 and 2017/2018 growing seasons from ten orchards in the Central Valley of Chile (Valparaíso, Metropolitan, and del Libertador Bernardo O’Higgins Regions) (Table 1).

The mycelia of 10 sensitive isolates, including the wild-type strain B05.10 and 22 isolates with reduced sensitivity to fludioxonil, were grown in yeast malt agar culture medium (malt extract 20 g/L, yeast extract 5 g/L, agar 12.5 g/L) and incubated at 20 °C with 80% of humidity for six to seven days in darkness until a mycelial layer with abundant sporulation was obtained. For gene expression analysis, the culture medium was supplemented with or without fludioxonil (Scholar^®^ 23% SC, Syngenta S.A., Monthey, Switzerland) at a 0.05 µg/mL concentration.

### 2.2. Assessment of Survival Structures Development 

The following fitness parameters were analyzed for the ten sensitive isolates and 12 isolates with reduced sensitivity to fludioxonil (11, 12, 13, 15, 16, 17, 18, 21, 22, 25, 26, and 27). Three replicates were performed per isolate.

Sclerotia and spore production in vitro: Petri dishes containing yeast malt agar culture medium (malt extract 20 g/L, yeast extract 5 g/L, agar 12.5 g/L) were inoculated with 20 µL of conidia suspension (10^5^ spores/mL). For sclerotia production, the number of sclerotia was evaluated after 40 days of incubation in continuous darkness at 5 °C and 20 °C and with 80% humidity. For spore production, the dishes were incubated for ten days in continuous darkness at 20 °C and 80% humidity. Sporulating colonies were rinsed with 20 mL of distilled sterile water, and the conidia suspension was filtered through 34 µm nylon mesh to remove fungal mycelium. The spore concentration in the suspension was estimated using a hemacytometer.

### 2.3. RNA Extraction, cDNA Synthesis, and Relative Quantification of atrB Gene Expression

From the mycelia biomass (100 mg) obtained from the 32 *B. cinerea* isolates treated with or without fludioxonil, the RNA was extracted using the RNeasy plant mini kit protocol (QIAGEN, Germantown, MD, USA). The RNA obtained from each isolate was dissolved in 35 µL of nanopure water and 1µL aliquot of this dilution was used for determining its concentration using an Epoch™ Microplate Spectrophotometer (BioTek, Winoosky, VT, USA). The RNA sample was adjusted to 1000 ng and treated with DNase for 20 min at 42 °C. The cDNA was synthesized using the Quantitect^®^ Reverse transcription kit (QIAGEN, Germantown, MD, USA) according to the manufacturer’s instructions. The program for the cDNA synthesis was 30 min at 42 °C followed by 3 min at 95 °C. For quantitative PCR analysis, two primer pairs were designed to amplify the target gene *atrB* and the reference gen *actin* (Table 2). The PCR was conducted using Brilliant III Ultra-Fast SYBR^®^ GREEN QPCR Master Mix (Agilent Technologies, Santa Clara, CA, USA) according to the manufacturer’s instructions. The amplification programs consisted of an initial denaturation at 95 °C for 3 min, followed by 50 cycles at 95 °C for 15 s, 55 °C for 15 s, and 72 °C for 15 s. A melting curve analysis was also performed to verify the specificity of amplified products. Then, the mRNA level was determined using the ΔΔCt method [44]. Values were normalized to the expression of the B05.10 strain. Three experiments with three biological replicates were conducted independently.

### 2.4. DNA Extraction, Amplification, and Sequencing of mrr1 Coding Region and atrB Intergenic and Coding Regions

From the biomass obtained from the 32 isolates of *B. cinerea*, 70–100 mg were recovered, and DNA was extracted using the DNeasy plant mini kit protocol (QIAGEN, Germantown, MD, USA). The DNA obtained from each isolate was dissolved in 50 µL of nanopure water (Promega, Madison, WI, USA) and a 1µL aliquot of this dilution was used for PCR amplification. From the sequence of the B05.10 strain (https://fungi.ensembl.org/Botrytis_cinerea/Info/Index database, accessed on 15 June 2022), a total of 15 pairs of primers were designed using the primer blast tool from NCBI (National Center for Biotechnology Information), six pairs for the amplification of the coding sequence of the *mrr1* gene and nine pairs for the amplification of the intergenic and coding sequence of *atrB* gene (Table 3). PCRs were performed in a total volume of 25 μL using the Go Taq^®^ Green Master Mix solution (Promega, Madison, WI, USA) according to the manufacturer’s instructions. The amplification program for the *atrB* and *mrr1* genes consisted of an initial denaturation at 95 °C for 3 min, followed by 40 cycles of 95 °C for 30 s, annealing temperature according to the primer for 30 s, and extension at 72 °C for 1 min and final step of 72 °C for 7 min. The amplified fragments were verified in a 2% agarose gel, stained with Red Gel^®^ (Biotium, Fremont, WA, USA), and a 100 bp ladder DNA length marker (Thermo Fisher Scientific Inc., Waltham, MA, USA) was used. All PCR products were sequenced by Psomagen USA Corp. (Rockville, MD, USA), and the DNA sequence analysis was performed using the software Vector NTI suite 7 [45]. 

The identification of the 5′UTR and 3′UTR sequences and the presence of introns in both genes was carried out using the sequence of the B05.10 strain from the https://fungi.ensembl.org/Botrytis_cinerea/Info/Index database (accessed on 15 June 2022). 

The functional analysis for identifying the functional domains of the Mrr1 transcription factor and the ABC transporter AtrB was performed using the InterPro tool [46].

### 2.5. Structural Alignment and Molecular Docking Simulations

Structural alignment was analyzed between the wild-type and mutated ABC transporter AtrB amino acid sequence and molecular docking of ATP with the wild-type and mutated ABC transporter AtrB amino acid sequence. The amino acid sequences of ABC transporter AtrB from the *B. cinerea* sequenced strain B05.10 were acquired from NCBI, and the amino acid sequences of the nine isolates (Genotype 3) with mutations in the ABC transporter AtrB were obtained using the software Vector NTI suite 7 [45].

The crystal structures for each ABC transporter AtrB amino acid sequence were obtained using the Phyre2 (Protein homology y recognition engine V 2.0) [47]. For the structural alignment between the crystal structures of wild-type and mutated isolates, the Multiseq-VMD (Visual Molecular Dynamics) was used, and the RMSD (Root Mean Square Deviation) value was calculated to determine differences between the structures [48]. The molecular docking was performed in a flexible manner of the molecule portions, which allowed the rotation around the degrees of freedom of the molecules and the calculation of free energy values between them. The setup of the structure was performed using Autodock Tools [49]; a docking area was defined by generating a box that encompassed the entire area of the protein, and Adenosine-5-triphosphate (ATP) was used as a ligand (PubChem CID 5957).

### 2.6. Statistical Analysis

Statistical analysis for sclerotia and spore production tests was conducted in InfoStat (version 2008) [50]. The evaluations were assessed using a one-way analysis of variance, and means were compared with Tukey’s protected least significant difference (*p* = 0.05).

## 3. Results

### 3.1. Botrytis Isolates with Reduced Sensitivity to Fludioxonil Presented Unaltered Survival Structure Production

**Figure 1:**
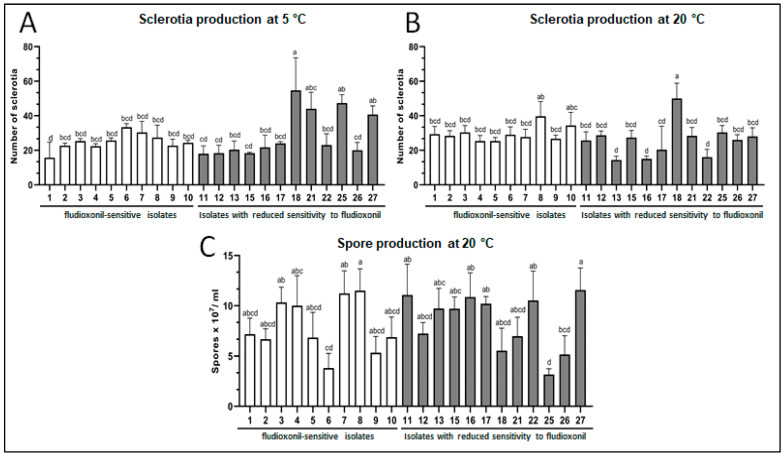
Survival structures in *Botrytis cinerea* isolates sensitive or with reduced sensitivity to fludioxonil. (**A**): Sclerotia production at 5 °C. (**B**): Sclerotia production at 20 °C. (**C**): Spore production at 20 °C. White bars: sensitive isolates. Gray bars: isolates with reduced sensitivity to fludioxonil. The bars with different letters a, b, c and d are significantly different (*p* < 0.05).

Many factors contribute to *Botrytis cinerea*’s success as a pathogen, including the prolific development of dispersal and survival structures. In contrast, isolates with reduced sensitivity to fludioxonil are infrequent within the Botrytis population [26]. We hypothesize defects in developing survival structures in Botrytis isolates with reduced sensitivity to fludioxonil, so we studied their conidia and sclerotia production. Sclerotia development was similar among all fludioxonil-sensitive isolates at a temperature of 5 °C; however, isolates with reduced sensitivity to fludioxonil showed either an equivalent (66.7%) or greater (33.3%) number of sclerotia than the sensitive population (Figure 1A). At 20 °C, two fludioxonil-sensitive isolates mostly induced sclerotia development. Isolates with reduced sensitivity to fludioxonil showed equivalent sclerotia development as 66.7% of the sensitive population (Figure 1B). However, three isolates exhibited a lower degree of sclerotia growth. Remarkably, an isolate with reduced sensitivity to fludioxonil (number 18) presented more sclerotia at 5 °C and 20 °C (Figure 1A,B). On the other hand, there were two categories within each population based on their conidia production. The first category included isolates that produce over 5.5 × 10^7^ conidia/mL, with 70% being sensitive to fludioxonil and 66.7% belonging to the reduced sensitivity to fludioxonil population. The remaining isolates were in the second category, characterized by a lower conidia production (Figure 1C). Our observations indicate that reduced sensitivity to fludioxonil in *Botrytis cinerea* isolates does not severely impact the development of survival structures. 

### 3.2. Botrytis cinerea Isolates with Reduced Sensitivity to Fludioxonil Exhibit Higher Endogenous Levels of atrB Expression

Quantitative real-time PCR was performed to investigate the *atrB* expression level in Chilean *B. cinerea* isolates that are sensitive or have a reduced sensitivity to fludioxonil. The isolates were grown in a fludioxonil-free medium or amended with 0.05 μg/mL of the fungicide. The experimental fungicide concentration chosen reduced mycelial growth in fludioxonil-sensitive isolates and had minimal effects on isolates with reduced sensitivity to fludioxonil (Supplemental Figure S1). The contrasting physiological response between the populations of both Botrytis isolates provides a valuable window to investigate the mechanisms of reduced-fludioxonil sensitivity.

**Figure 2:**
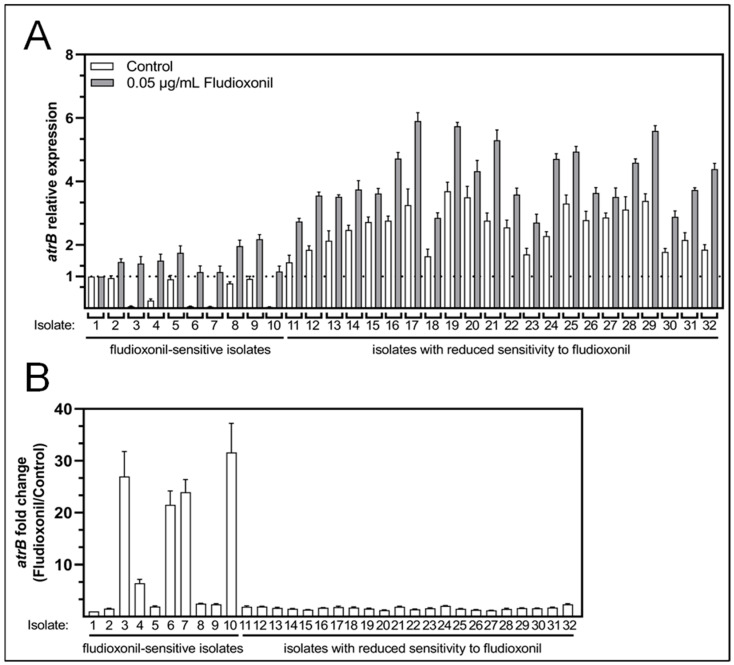
Relative expression of the *atrB* gene of *B. cinerea* isolates used in this study. (**A**): The *atrB* relative expression was normalized with the *actin* gene expression and then calibrated to the values of mRNA amount based on the isolate with the lowest amount of *atrB* mRNA (B05.10). White bars: sensitive and with reduced sensitivity to fludioxonil isolates without fludioxonil treatment. Gray bars: sensitive and reduced sensitivity to fludioxonil isolates with 0.05 µg/mL of fludioxonil treatment. (**B**): *atrB* fold change of sensitivity and with reduced sensitivity to fludioxonil isolates.

Two *atrB* expression patterns were observed in susceptible isolates in the absence of fludioxonil. Fifty percent of fludioxonil-sensitive isolates presented *atrB* levels close to the housekeeping gene expression, while the remaining fifty percent presented levels close to zero (Figure 2A). Remarkably, all isolates with reduced sensitivity to fludioxonil exhibited endogenous *atrB* overexpression (Figure 2A). Among these isolates, 23% (isolates 11, 18, 23, 30, and 32) presented the lowest *atrB* overexpression, 1.5- and 2-fold over the *atrB* expression in fludioxonil-sensitive isolates. Two isolates (9%; isolates 19 and 20) have the highest *atrB* levels, corresponding to 4-fold over the fludioxonil-sensitive isolates *atrB* expression level. The remaining 68% of the population presented intermediate overexpression levels in the range of 2.5- and 3-fold over the fludioxonil-sensitive isolates *atrB* expression level (Figure 2A). Supplementation of the growth medium with fludioxonil (0.05 μg/mL) increased *atrB* expression in both sensitive isolates and isolates with reduced sensitivity to fludioxonil compared to their endogenous *atrB* levels (Figure 2A). Fludioxonil increased *atrB* expression more significantly than the reference expression value in all fludioxonil-sensitive isolates, with an average of 20-fold induction in 50% of these isolates (Figure 2B). While fludioxonil-mediated induction of *atrB* expression in *B. cinerea* isolates with reduced sensitivity to fludioxonil was 1- to 2-fold the endogenous *atrB* expression level recorded for these isolates (Figure 2B). The results indicate that the expression levels of *atrB* are higher in the *Botrytis cinerea* population with reduced sensitivity to fludioxonil compared to the sensitive population. Also, fludioxonil-mediated induction of *atrB* expression depends on the sensitivity background.

### 3.3. B. cinerea Isolates with Reduced Sensitivity to Fludioxonil Carry a Unique mrr1 Version Divergent from Fludioxonil-Sensitive Isolates

**Figure 3:**
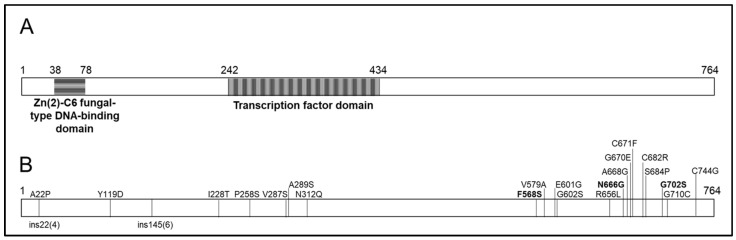
(**A**): Functional domains present in the Mrr1 transcription factor: the Zn(2)-C6 fungal-type DNA-binding domain and the transcription factor domain. (**B**): Detected amino acid changes in *Botrytis cinerea* isolates with reduced sensitivity to fludioxonil. The mutations previously reported are indicated in bold letters.

The complete coding sequence of the *mrr1* gene of the 32 *B. cinerea* isolates was amplified using the primers described in Table 3. Six fragments were assembled to obtain a coding sequence of 2444 bp. Two 73 bp and 75 bp introns were identified between positions 185–258 and 1114–1190, respectively. In addition, 5′UTR and 3′UTR sequences of 366 bp and 1179 bp, respectively, were also recognized. The genomic *mrr1* region of the 32 Chilean isolates under study was compared to the B05.10 strain’s reference sequence. Only two genotypes were observed. The *mrr1* sequence found in the fludioxonil-sensitive isolates was identical to that of B05.10 (Genotype 1). In contrast, the 22 isolates with reduced sensitivity to fludioxonil presented a mutated, conserved, and divergent version of *mrr1* regarding fludioxonil-sensitive isolates (Genotype 2). Genotype 2 presented thirty-two changes in the coding region, of which two corresponded to insertions, nine to synonymous mutations, and twenty-one to no synonymous mutations (Figure 3B). The Mrr1 transcription factor sequence has been analyzed bioinformatically, exhibiting two distinct protein domains (Figure 3A). The first domain is the Zn(2)-C6 fungal-type DNA-binding domain, which spans from residue 38 to residue 78 and presents amino acid identity between sensitive and with reduced sensitivity to fludioxonil isolates. The second domain is the transcription factor domain, which spans from residue 242 to residue 434 and has four out of the twenty-one observed non-synonymous mutations (P258S, V287S, A289S, N312Q) concentrated within it (Figure 3B). Of the remaining seventeen non-synonymous amino acid changes, three were concentrated within the N-terminus and fourteen in the C-terminus of the protein (Figure 3B).

### 3.4. High Variability in atrB Does Not Correlate with Changes in the Function of the ABC Transporter AtrB

The genomic sequence of the *atrB* gene from 32 isolates of *B. cinerea* was amplified employing the primers described in Table 3. After analyzing the assembled fragment, we found that all isolates have a consistent gene configuration, which includes an 1802 bp intergenic region sequence (putative promoter region) and a 4329 bp coding region sequence. An intron of 56 bp between positions 1115 and 1171 was also observed. The 5′ UTR and 3′UTR sequences of 151 bp and 338 bp, respectively, were also recognized. To explore mutations in the *atrB* genomic sequence, we compared the complete *atrB* from the 32 *B. cinerea* field isolates under study with the reference *atrB* from the *B. cinerea* B05.10 strain. Four different *atrB* genotypes were found, and they were not linked to sensitivity to fludioxonil. Five sensitive isolates and seven with reduced sensitivity to fludioxonil presented Genotype 1, characterized by six nucleotide mutations in the intergenic region and one silent mutation in the coding region. Four sensitive isolates and two with reduced sensitivity to fludioxonil presented Genotype 2, with twelve nucleotide mutations in the intergenic region and three silent mutations in the coding region of the *atrB*. Nine isolates with reduced sensitivity to fludioxonil presented Genotype 3, represented by 14 nucleotide mutations in the coding region of the *atrB* gene. Among these changes, five resulted in amino acid mutations (N715H, Y737H, A763S, S775A, and E797G), and the remaining nine were silent mutations. Finally, four isolates with reduced sensitivity to fludioxonil presented Genotype 4 with 18 nucleotide mutations in the intergenic region and 9 silent mutations in the coding region of *atrB* (Table 4).

Only Genotype 3 presented non-synonymous amino acid changes in the *atrB* coding region. Notably, Genotype 3 includes only isolates with reduced sensitivity to fludioxonil. Therefore, we explore the impact of the amino acid changes on the ABC transporter AtrB function. Through the bioinformatics analysis of the ABC transporter AtrB, four functional domains were identified: two nucleotide-binding domains (NBDs) and two transmembrane (transporter) domains (TMDs). The five mutations of Genotype 3 localized in the NBD of the ABC transporter AtrB. Therefore, we asked whether these aminoacidic changes modified the nucleotide-binding of ABC transporter AtrB. To evaluate this hypothesis, we contrasted the crystal structures of Genotype 3 ABC transporter AtrB with the wild-type version using a structural alignment.

The results showed an RMSD value of 0.58234 Å; this low value suggests the absence of a conformational change associated with the detected mutations. This result was confirmed by performing molecular docking, where the efficiency parameters obtained were identical between the wild-type protein-ATP and the mutated protein-ATP (Table 5).

## 4. Discussion

Fludioxonil has been widely used pre- and post-harvest in Chile since the 1990s. It has been characterized for its high effectiveness in controlling *B. cinerea* populations in several crops. However, during the 2016/2017 and 2017/2018 seasons, the first isolates with reduced sensitivity to the molecule were detected in Chilean table grape fields with a history of fludioxonil application associated with the cyprodinil & fludioxonil mixture [41]. The Chilean isolates with fludioxonil EC_50_ values up to 2.4 μg/mL were categorized as low to moderately resistant [38]. This result is consistent with worldwide reports, indicating the absence of isolates recovered from the field highly resistant to fludioxonil (EC_50_ > 5 μg/mL) [34,37,39]. However, unique isolates highly resistant to fludioxonil have been detected in some cases, from strawberries, pumpkins, tomatoes, and cut flowers grown in greenhouses, where favorable conditions exist for fungus development [26,27,51,52]. The frequency of isolates with some level of fludioxonil resistance, detected in Chile and other parts of the world, remains low [26,37,41]. Alterations on the Group III hybrid histidine kinase signaling support fludioxonil resistance. These histidine kinases are involved in adaptation to adverse environmental conditions such as osmotic and fungicide stresses and, in addition, play an essential role in the development and pathogenesis [53,54,55]. In contrast, Chilean isolates with reduced sensitivity to fludioxonil did not present fitness cost or aggressiveness alterations regarding sensitive isolates to the fungicide [41]. The survival structures evaluation in this study indicated a similar propagation potential for the *B. cinerea* population with reduced sensitivity to fludioxonil relative to sensitive isolates. Based on the available data, there appears to be a mechanism of fludioxonil tolerance unrelated to energy overuse in Chilean isolates. Alternatively, the overexpression of Multidrug Resistance transporters such as ABC transporter AtrB may explain fludioxonil tolerance without affecting the overall *B. cinerea* fitness [34]. Our analysis revealed higher *atrB* endogenous levels in isolates with reduced sensitivity to fludioxonil and lower induction of *atrB* expression triggered by the fungicide regarding sensitive isolates. The fludioxonil-induced induction of *atrB* is significantly more abrupt in fludioxonil-sensitive isolates. In 50% of the sensitive population, fludioxonil induces 20-fold higher levels than the control, while isolates with reduced sensitivity show a maximum of 3-fold induction levels. This suggests that the energetic cost makes it impossible for sensitive isolates to survive over time. In addition, the highest level of transcripts obtained by isolates with reduced sensitivity to fludioxonil are consistent with different reports [37,38]. Also, the *atrB* expression levels did not correlate with the mutations detected in its regulatory region, as isolates with the same mutations displayed inconsistent sensitivity to fludioxonil. Therefore, it is possible that *B. cinerea* isolates from Chile exhibit reduced sensitivity to fludioxonil due to additional cellular bases independent of *atrB* regulation.

The *atrB* regulation depends on the transcription factor Mrr1. Different authors have indicated that isolates with mutant versions of Mrr1 present alterations in sensitivity to fludioxonil, giving rise to isolates that are low and moderately resistant to the molecule [34,36,37,38]. Chilean isolates with reduced sensitivity to fludioxonil consistently presented a divergent version of Mrr1 compared to the wild-type protein. We found over thirty coding sequence changes, of which three have been previously detected by other authors (F568S, N666G, and G702S) [37,38]. The quantity of mutations is surprisingly elevated and mainly focused on the protein’s C-terminal region and the transcription factor domain. Therefore, this suggests that the mutations present in the transcription factor domain could be associated with the increase in the level of *atrB* transcripts in isolates with reduced sensitivity to fludioxonil without ruling out the hypothesis that there may be other relevant transcriptional targets or protein partners that account, for example, for their survival capacity. Remarkably, there is no clear correlation between the overexpression of *atrB* and the absence of mutations in Mrr1 in Botrytis fragariae, suggesting alternative mechanisms may be involved in regulation of *atrB* expression [56]. In addition, this study did not detect the deletion of three base pairs, which led to the deletion of L497 in Mrr1. This deletion is associated with the MDR1h phenotype, and it is found in isolates with higher levels of fludioxonil resistance (EC_50_ > 3.1 μg/mL) than those analyzed in this study [38,39]. The significant variation in Mrr1 could be attributed to the high fungicide selection pressure on the *B. cinerea* populations because various fungicides employed in the phytosanitary programs are detoxified by the ABC transporter AtrB, including anilinopyrimidines, dicarboxamides, benzimidazoles and other fungicide families [34].

Finally, we determined that direct changes in the ABC transporter AtrB amino acid sequence are unrelated to modifications in sensitivity to fludioxonil. Only a subset of isolates with reduced sensitivity to fludioxonil exhibited non-synonymous alterations in the ABC transporter AtrB. We compared the crystal wild-type and mutated ABC transporter AtrB and calculated the RMSD value to determine whether the mutations altered the protein structure. RMSD value is considered a reliable indicator of variability when applied to similar proteins, like alternative conformations of the same protein. The RMSD is 0 for identical structures, which increases as the two structures become more different [57]. In this study, the RMSD value was approximately 0.5 Å, a value categorized as low, suggesting that the five mutations detected in the ABC transporter AtrB of isolates with reduced sensitivity to fludioxonil did not alter the structural conformation of the protein. Therefore, we improved our analysis by studying the docking of ATP with both wild-type and mutant versions of the protein. We performed two molecular dockings: wild-type NBDs protein and mutated NBDs protein, with ATP molecules as ligands. The NBDs consist of a RecA-type ATP-binding core that is supplemented with an ABC-specific three-stranded antiparallel β-sheet (ABCβ) and a nα-helical subdomain (ABCα) [58]. The result showed the same efficiency parameters in both conditions evaluated, demonstrating that the physicochemical properties of the mutated amino acids present in the NBD do not alter the ATP union. According to our evaluation parameters, no changes were observed in the ABC transporter AtrB. This suggests that the mutations did not affect its functionality and the required energy produced to excrete the fungicide outside the cell. 

## 5. Conclusions

*Botrytis cinerea* isolates with reduced sensitivity to fludioxonil are emerging in table grapes in Chilean Central Valley fields. However, fludioxonil is still a valuable tool for effective grey mold control. This study found that all Chilean isolates with reduced sensitivity to fludioxonil consistently carry a version of the Mrr1 transcription factor with 21 non-synonymous changes and two deletions. Furthermore, the isolates exhibit a significant level of *atrB* expression, which is the specific target of Mrr1. The C-terminal portion of the protein harbors most of the changes in Mrr1, indicating modifications in its regulation or transcriptional partners may have occurred for isolates with reduced sensitivity to fludioxonil. Reduced sensitivity to fludioxonil may be due to changes in the regulation of Mrr1 transcriptional targets, resulting in low to no fitness cost for isolates. Although isolates with reduced sensitivity to fludioxonil are infrequent, it is essential to implement anti-resistance measures in the field due to their high efficacy and the development of new mixtures that include this molecule as an active ingredient.

## Figures and Tables

**Table 1:** List of *Botrytis cinerea* isolates used in this study.

N° of Isolates	Nomenclature **	Region	Season	EC_50_ Fludioxonil (μg/mL)	Sensitivity to Fludioxonil
1 *	-	-	-	0.01	S
2	FBB7 9A3	Valparaíso	2017/2018	0.011	S
3	FCF6 41B1	Valparaíso	2016/2017	0.013	S
4	FBF7 10A2	Valparaíso	2017/2018	0.014	S
5	FBF7 10A3	Valparaíso	2017/2018	0.016	S
6	FCF7 44D1	Valparaíso	2017/2018	0.022	S
7	FCF7 44D2	Valparaíso	2017/2018	0.026	S
8	FCF7 45B1	De O’Higgins	2017/2018	0.045	S
9	FCF7 45B2	Valparaíso	2017/2018	0.048	S
10	FCF7 45B3	Metropolitan	2017/2018	0.055	S
11	FBF6 18B4	Valparaíso	2016/2017	1.08	R
12	FBF6 11B4	Valparaíso	2016/2017	1.09	R
13	FBF6 18B3	Valparaíso	2016/2017	1.10	R
14	FBF7 7B10	Valparaíso	2017/2018	1.11	R
15	FBF6 12A3	Valparaíso	2016/2017	1.14	R
16	FBF6 18B1	De O’Higgins	2016/2017	1.17	R
17	FBF6 12C1	Valparaíso	2016/2017	1.19	R
18	FDF6 33 C2	Valparaíso	2016/2017	1.22	R
19	FBF7 7B6	Valparaíso	2017/2018	1.24	R
20	FBF7 8D1	Valparaíso	2017/2018	1.25	R
21	FBF6 14C2	Valparaíso	2016/2017	1.30	R
22	FBF6 11B3	Valparaíso	2016/2017	1.37	R
23	FBF7 7B2	Valparaíso	2017/2018	1.44	R
24	FBF7 8B2	Valparaíso	2017/2018	1.46	R
25	FBF6 15B3	Valparaíso	2016/2017	1.47	R
26	FBF6 11C2	Valparaíso	2016/2017	1.50	R
27	FBF6 15B1	Valparaíso	2016/2017	1.51	R
28	FBF7 7B1	Valparaíso	2017/2018	1.68	R
29	FBF7 8B3	Valparaíso	2017/2018	2.04	R
30	FBF7 6B3	Valparaíso	2017/2018	2.21	R
31	FBF7 6A1	Valparaíso	2017/2018	2.22	R
32	FBF7 6C1	Valparaíso	2017/2018	2.37	R

* B05.10 strain used as wild-type; **: nomenclature used in previous study [41]; S: sensitive; R: resistant.

**Table 2:** List of primers for amplification of partial *atrB* cDNA and partial *actin* cDNA using qPCR.

Primer Name	Sequence 5′->3′	T° Annealing (°C)	Fragment Size (bp)
BcatrB_qPCR_F	ACGTTTGACAATTGGTGTGG	55	150
BcatrB_qPCR_R	GAGTTGAGCGGAAGGTTGAT	55	150
Bcact_qPCR_F	CTGGTCGTGATTTGACTGATTA	55	100
Bcact_qPCR_R	GATTGACTGGCGGTTTGG	55	100

**Table 3:** List of PCR primers for amplifying intergenic and coding region of *atrB* gene (BcartB) and coding region of *mrr1* gene (Bcmrr1).

Primer Name	Sequence 5′->3′	T° Annealing (°C)	Fragment Size (bp)
BcartB_1F	ATGAGCTACACAGGGATCGAA	60	913
BcartB_1R	CGCAGTTTGTCAAAGCCGTC	60	913
BcartB_2F	AGTATGGATTCGGGCGAGTG	56	1125
BcartB_2R	TGTGGTCGAACAATCGAGGA	56	1125
BcartB_3F	CATCAAGGGGTTTCCGGTT	60	978
BcartB_3R	GAAACACGCTTACGCTCACC	60	978
BcartB_4F	AGCACACCAATACCGAGGAC	51	843
BcartB_4R	CGCAAGCTTTGACTTGGGTC	51	843
BcartB_5F	CTACCACCGCCATCGCTAAA	60	782
BcartB_5R	CACAAGCTTGGAACGCCAAA	60	782
BcartB_6F	CTTGCCTATGGCTTTTCGGC	54	779
BcartB_6R	GTCGGGGACGGTTCTTGATT	54	779
BcartB_7F	AAGCCGGGTATGTTAGGTGC	61	964
BcartB_7R	AGACCGCCGACTGAATGTTT	61	964
BcartB_8F	CTGGCTCAACTCTCCCGAAT	53	794
BcartB_8R	TACTCGCCACAAGTGCCATT	53	794
BcartB_9F	CCTCGTCTTCACCAGTTGGG	63	842
BcartB_9R	TCTTGGGGATTTGCCGATGT	63	842
Bcmrr1_1F	GGATGCGGATGGTTACGGAT	60	1057
Bcmrr1_1R	TGATGGCGGATTTGACCGAA	60	1057
Bcmrr1_2F	TAATCAGGCATCTGGCACGG	62	921
Bcmrr1_2R	CGATCATGAGTGCGCATAGC	62	921
Bcmrr1_3F	GTCTCGAGGCTAGCGTGTTT	63	938
Bcmrr1_3R	AACCGCAATTACATGCCACG	63	938
Bcmrr1_4F	CTCGAGGACAATCCTTGCGT	62	867
Bcmrr1_4R	GTGCTTGCTTGAAAGTGCGA	62	867
Bcmrr1_5F	AGTTTGTAGCGATGGACCCC	60	1203
Bcmrr1_5R	CGTCAGTGTCGCCCAGAATA	60	1203
Bcmrr1_6F	ACGGAATCGATGCCTGTGAA	60	959
Bcmrr1_6R	ATATCTGCGGCAGCCTTGAG	60	959

**Table 4:** Nucleotide mutations in the intergenic and coding region of the *atrB* gene, aminoacidic mutations in the ABC transporter AtrB, and the genotype number of the 32 isolates of *B. cinerea*.

N° of Isolates	EC_50_ Fludioxonil (μg/mL)	Sensitivity to Fludioxonil	Nucleotide Changes in the Intergenic Region	Nucleotide Changes in the Coding Region	Amino acid Changes	Genotype
2	0.011	sensitive	T-490C; A-858G; C-1259T; T-1614C; A-1748G; T-1757C	C3591T	N.D.	1
4	0.014	sensitive				
5	0.016	sensitive				
8	0.045	sensitive				
9	0.048	sensitive				
12	1.09	resistant				
16	1.17	resistant				
17	1.19	resistant				
19	1.24	resistant				
26	1.50	resistant				
28	1.68	resistant				
29	2.04	resistant				
3	0.013	sensitive	C-67T; G-68T; G-413A; T-490C; G-710A; A-858G; C-1259T; T-1694G; A-1748G; C-1756T; T-1757C; C-1779T	A369G; A1200G; T3675C	N.D.	2
6	0.022	sensitive				
7	0.026	sensitive				
10	0.055	sensitive				
11	1.08	resistant				
18	1.22	resistant				
13	1.10	resistant	N.D	T1149C; A1200G; A1645G; **A2143C**; C2169T; **T2209C**; **G2287T**; **T2323G**; C2325T; C2379T; **A2390G**; C2457T; G2479A; T3675C	N715H; Y737H; A763S; S775A; E797G	3
14	1.11	resistant				
15	1.14	resistant				
20	1.25	resistant				
21	1.30	resistant				
22	1.37	resistant				
23	1.44	resistant				
24	1.46	resistant				
25	1.47	resistant				
27	1.51	resistant	A-54G; T-187G; T-252C; C-256T; G-631T; A-858G; A-1172T; C-1259T; A-1313G; C-1378T; A-1410C; A-1413G; G-1419T; A-1603T; A-1748G; T-1757C; T-1769C; G-1782C	A684T; A921C; T1149C; A1200G; T2316C; C2325T; T3675C; C3768T; A3915G	N.D.	4
30	2.21	resistant				
31	2.22	resistant				
32	2.37	resistant				

N.D: No Detection, Mutations in bold: associated with amino acid changes.

**Table 5:** Efficiency parameters obtained from molecular docking ABC transporter AtrB-ATP. WTP-ATP: Wil-type Protein-ATP. MP-ATP: Mutated Protein-ATP.

	Efficiency Parameters (Kcal/mol)					
	Binding Energy	Ligand Efficiency	Intermolar Energy	Electrostatic Energy	Torsional Energy	Free Energy
WTP-ATP	0.97	0.03	3.5	1.97	4.47	7.13
MP-ATP	0.97	0.03	3.5	1.97	4.47	7.13

## Data Availability

Data are the property of the Laboratory of Fruit and Molecular Phytopathology of Faculty of Agronomic Sciences, University of Chile, Santiago, Chile. Any requests should be directed to Marcela Esterio (Principal Investigator).

## Supplementary Materials

The following supporting information can be downloaded at: https://www.mdpi.com/article/10.3390/pathogens13050374/s1, Figure S1: Mycelial growth of Botrytis isolates subjected to different concentrations of fludioxonil. The graph shows the mycelial growth of three representative isolates sensitive to fludioxonil (green lines) and three isolates presenting reduced sensitivity to fludioxonil (red lines). The concentrations of fludioxonil correspond to those used to calculate the EC_50_ value.
