# Supplementary material for: A Conservative Mutant Version of the Mrr1 Transcription Factor Correlates with Reduced Sensitivity to Fludioxonil in Botrytis cinerea
Source: Pathogens. 2024 Apr 30;13(5):374. doi: 10.3390/pathogens13050374 (PMC11124108; doi:10.3390/pathogens13050374)
Supplement: Supplementary file 1 [file pathogens-13-00374-s001.zip › pathogens-2784215-supplementary.pdf]

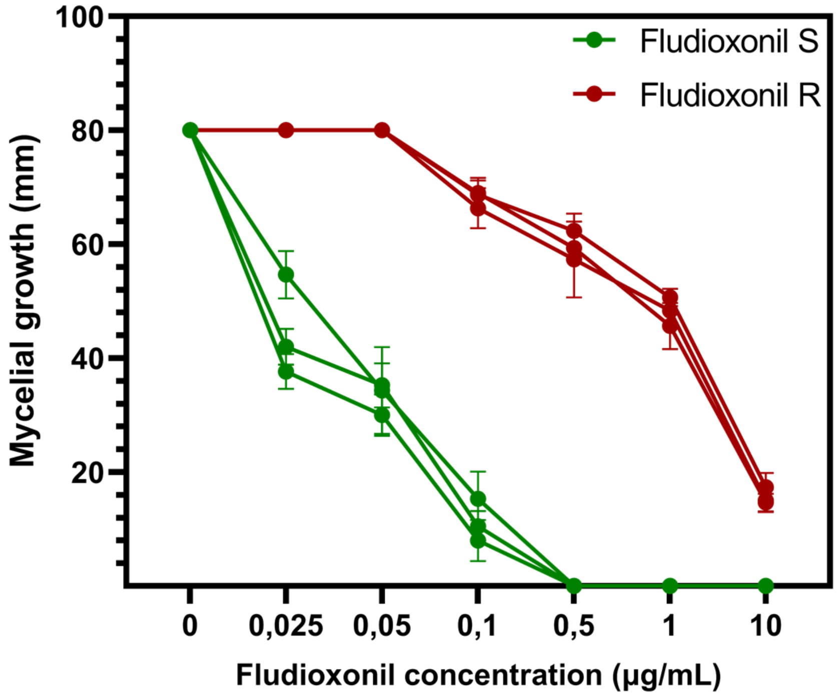

Supplemental Figure S1. Mycelial growth of Botrytis isolates subjected to different concentrations of fludioxonil. The graph shows the mycelial growth of three representative isolates sensitive to fludioxonil (green lines) and three isolates presenting loss of sensitivity to fludioxonil (red lines). The concentrations of fludioxonil correspond to those used to calculate the EC_{50} value.
